# Evaluation of gastroprotective and ulcer healing activities of yellow mombin juice from *Spondias mombin* L.

**DOI:** 10.1371/journal.pone.0201561

**Published:** 2018-11-05

**Authors:** Samara A. Brito, Isabela S. Barbosa, Cynthia L. F. de Almeida, Jonathan W. de Medeiros, Jacinto C. Silva Neto, Larissa A. Rolim, Teresinha G. da Silva, Rafael M. Ximenes, Irwin R. A. de Menezes, Germana F. R. Caldas, Almir G. Wanderley

**Affiliations:** 1 Department of Pharmaceutical Sciences, Universidade Federal de Pernambuco, Recife, Pernambuco, Brazil; 2 College Santa Maria, Cajazeiras, Paraíba, Brazil; 3 Department of Physiology and Pharmacology, Universidade Federal de Pernambuco, Recife, Pernambuco, Brazil; 4 College of Nursing, Universidade Federal do Vale de São Francisco, Petrolina, Pernambuco, Brazil; 5 Department of Cellular and Applied Molecular Biology, Universidade Estadual de Pernambuco, Recife, Pernambuco, Brazil; 6 Department of Histology and Embryology, Universidade Federal de Pernambuco, Recife, Pernambuco, Brazil; 7 Analytical Center of Drugs, Medicines and Food, Universidade Federal do Vale de São Francisco, Petrolina, Pernambuco, Brazil; 8 Department of Antibiotics, Universidade Federal de, Recife, Pernambuco, Brazil; 9 Department of Biological Chemistry, Universidade Regional do Cariri, Crato, Ceará, Brazil; 10 Centro Universitário Doutor Leão Sampaio, Juazeiro do Norte, Ceará, Brazil; Jagiellonian University Medical College, POLAND

## Abstract

*Spondias mombin* L. (yellow mombin) is a tree with a nutritional fruit that is commonly consumed in the North and Northeast of Brazil, as the juice of its pulp is rich in antioxidant compounds. This study aimed to investigate the gastroprotective and ulcer healing activities of yellow mombin juice (YMJ) in Wistar rats, and to elucidate the possible involved mechanisms. Phytochemical characterization of the lyophilized fruit juice was performed by high-performance liquid chromatography (HPLC). The gastroprotective activity of YMJ was investigated in ethanol (25, 50, and 100% YMJ) and indomethacin (100% YMJ) models of acute gastric ulcer in rats. Then, the effect of YMJ on mucus production and gastric secretions, and the involvement of non-protein sulfhydryl groups and prostaglandins in the gastroprotective process were examined. Moreover, the ulcer healing effect of YMJ was investigated in a model of acetic acid-induced chronic ulcer through histological and immunohistochemical analyses. HPLC results identified the presence of epicatechin (7.1 ± 1.6 μg/mL) and quercetin (17.3 ± 2.5 μg/mL) in YMJ. Ethanol-induced gastric lesions were inhibited by YMJ (25, 50, and 100%) by 42.42, 45.09, and 98.21% respectively, and indomethacin-induced lesions were inhibited by YMJ (100%) by 58.96%, compared to control group. Moreover, YMJ reduced gastric content and total acidy by 57.35 and 71.97%, respectively, compared to the control group. Treatment with YMJ also promoted healing of chronic ulcer, regeneration of the gastric mucosa, and restoration of mucus levels in glandular cells, as confirmed by histological analysis. It also increased cellular proliferation, as demonstrated by high reactivity to Ki-67 and bromodeoxyuridine. In conclusion, YMJ was found to possess gastroprotective and ulcer healing activities that are correlated to its antisecretory action. These results support the commercial exploration of YMJ as a functional food.

## Introduction

Peptic ulcer is a widespread disease whose incidence varies from one country to another, but is generally considered as a worldwide public health problem. Peptic ulcer is usually aggravated by an imbalance between destructive and defensive factors such as mucosal blood flow and production of mucus, bicarbonate, and prostaglandins in the stomach [1, 2]. Other factors may also trigger the development of peptic ulcers, such as *Helicobacter pylori* infection [3], ethanol consumption, prolonged use of non-steroidal anti-inflammatory drugs (NSAIDs), some dietary habits, and psychological stress [4]. On the other hand, gaseous mediators [5] and nesfatin-1 [6] were reported to be involved in acceleration of ulcer healing.

The genus *Spondias* (Anacardiaceae) comprises 18 species and several hybrids distributed in the Americas, Asia, and Madagascar. In tropical America, four *Spondias* species are of economic importance: *S*. *dulcis* Parkinson, *S*. *purpurea* L., *S*. *tuberosa* Arruda, and *S*. *mombin* L. The fruits of *S*. *mombin* L. are known by many different popular names, the best known of which in Brazil are cajá, cajá-mirim, and taperebá [7, 8].

Our research group when evaluating the antiulcerogenic capacity of the Spondias mombin ethanolic extract (SmEE) showed that SmEE has antiulcerogenic activity mediated by antioxidant activity, stimulation of the gastric mucus production or involvement of the sulfhydryl groups and nitric oxide, besides antisecretory, anti-Helicobacter pylori activities and cicatrizante gástrico. In addition, they found that the major compounds found in SmEE, ellagic acid and gallic acid are gastric protectors and when evaluated in association the protective activity is increased [9].

*S*. *mombin* (yellow mombin) fruits play an important role in the agribusiness of the North and Northeast regions of Brazil due to their characteristic flavor and exotic aroma. They are popularly consumed either as fresh fruits or processed pulp, juices, or ice cream [10]. The fruit contains phenolic compounds and thus, exhibits antioxidant activities [11], and can reduce inflammation and lipid peroxidation [12] that take place in gastric lesions.

There is a growing interest in functional foods containing phytochemicals, which are non-nutritive plant chemicals that can be applied in the prevention or treatment of diseases [13]. In this study, we aimed to investigate the gastroprotective and ulcer healing properties of yellow mombin juice (YMJ), and to elucidate the mechanisms of action involved in these effects.

## Material and methods

### Reagents and chemicals

The following substances were used in our study: Alcian Blue, N-ethylmaleimide (NEM), nitro-L-arginine methyl ester, ranitidine, carbenoxolone (Sigma-Aldrich, St. Louis, USA), acetic acid, glucose (Vetec, Duque de Caxias, Brazil), ethyl ether, formaldehyde, phenolphthalein (FMaia, Cotia, Brazil), xylazine, ketamine (Vetbrands, Paulinia, Brazil), Ki-67 antibody (code: sc-23900), and bromodeoxyuridine (BrdU) antibody (code: sc-32323) (Santa Cruz Biotechnology, Santa Cruz, CA, USA).

### Plant material and extract preparation

*S*. *mombin* fruits were collected in Crato, Ceará, Brazil, in December 2015 (S 7°13.00.6”–W 39°22.15.1”). A voucher specimen was deposited in the Herbarium of the Agronomic Institute under the registration number #91073. Botanical identification was done by Rita de Cássia Pereira. The pure fruit juice (5000 g) was dehydrated with the aid of a lyophilizer for phytochemical analysis and then dissolved and diluted in 0.9% NaCl solution to obtain 100, 50, and 25% solutions before being investigated in an *in vivo* experiment of ethanol-induced ulcer at a dose of 10 mL/kg. The pH of YMJ (2.79 ± 0.05) was determined in triplicate using a pH meter (Bante Instruments, Shanghai, China).

### Phytochemical study

Phytochemical analysis of YMJ was performed using high-performance liquid chromatography (HPLC) (Shimadzu, Kyoto, Japan) with a diode array detector (DAD), a C_18_ column with particle size of 5 μm and dimensions of 250 × 4.6 mm, and a guard column (Thermo Fisher Scientific, MA, USA). Temperature was maintained at 40 °C throughout the analysis. Two solutions were used for the mobile phase: Solution A, which consisted of phosphoric acid in ultra-purified water (0.1% v/v); and solution B, which consisted of acetonitrile (100%). The two solutions were delivered using a gradient system as follows: 0–60 min/95–5% (A), 60–70 min/5–95% (A), and 70–75 min/95% (A), at a flow rate of 0.6 mL/min. A volume of 40 μL of the analytical standards and samples was injected, and detection was performed by the DAD at a wavelength of 340 nm.

### Animals

Totally, 182 Wistar rats (200–320 g) of both sexes, obtained from the Department of Physiology and Pharmacology at the Federal University of Pernambuco (UFPE, Pernambuco, Brazil), were used in the experiments. Animals were kept under standard environmental conditions (12:12 h light-dark cycle, a temperature of 22 ± 2 °C, and a humidity of 55–65%), with free access to filtered water and industrialized dry food (Presence, Purina, São Paulo, Brazil). All experimental protocols were conducted in accordance with the *Guide for the Care and Use of Laboratory Animals* by the National Institute of Health (Washington, DC, 2011) and were submitted to and approved by the UFPE Ethics Committee on Animal Use, under license number 23076.019290/2016-52. In all experiments, animals were euthanized by CO_2_ inhalation in a CO_2_ chamber.

### Investigating the gastroprotective activity of YMJ

#### Ethanol-induced gastric lesions

After fasting for 16 h, rats were randomly distributed into five groups (n = 6/group) as follows: control group, rats received 0.9% NaCl by oral route; lansoprazole, rats were orally pretreated with lansoprazole (30 mg/kg); YMJ (25, 50, and 100), rats were orally pretreated with 25, 50, and 100% YMJ (10 mL/kg), respectively.

One hour after pretreatment, all animals received absolute ethanol (99.8%, 4 mL/kg) by oral administration to induce gastric lesions according to the method described by Morimoto et al. [14] with slight modifications. After 1 h, animals were euthanized, stomachs were removed and photographed, and lesions were measured (Image J software, Bethesda, MD, USA). Results were expressed as the total area of ulcerative lesions (mm^2^).

#### Indomethacin-induced gastric lesions

After fasting for 16 h, rats were randomly distributed into three groups (n = 6-8/group) as follows: control group, rats received 0.9% NaCl by oral route; ranitidine, rats were orally pretreated with ranitidine (60 mg/kg); YMJ (100), rats were orally pretreated with 100% YMJ (10 mL/kg). Thirty minutes after pretreatment, all animals received indomethacin (30 mg/kg) by subcutaneous administration to induce gastric lesions according to the method described by Djahanguiri [15]. After 6 h, animals were euthanized, stomachs were removed and photographed, and gastric lesions were evaluated as described previously.

### Evaluation of mucosal protective factors

#### Determination of mucus adhering to the gastric mucosa

This experiment was performed according to the method described by Raffatullah et al. [16] with some modifications. After fasting for 18 h, rats were randomly distributed into three groups (n = 6/group) as follows: control group, rats received 0.9% NaCl (1 mL/kg) by oral route; carbenoxolone, rats were orally administered carbenoxolone (200 mg/kg); and YMJ (100), rats were orally administered 100% YMJ (10 mL/kg). After 1 h, rats were anesthetized by an intraperitoneal (i.p.) injection of xylazine (6 mg/kg) and ketamine (60 mg/kg), then they were submitted to longitudinal incision for pylorus ligation. Four hours later, animals were euthanized, and the glandular portion of the stomach was separated, weighed, and immersed in 10 mL of 0.1% Alcian Blue solution (0.16 M sucrose/0.05 M sodium acetate, pH 5.8). After 2 h of immersion, excess dye was removed by rinsing with 7 mL of 0.25 M sucrose for two successive times. Each stomach was sequentially transferred to 10 mL of 0.5 M MgCl_2_ solution for 2 h. Then, 4 mL of the dye solution was shaken vigorously with an equal volume of ether. The resulting emulsion was centrifuged at 176 × *g* for 10 min, and the absorbance of the aqueous layer was measured at 595 nm. Then, the amount of blue dye extracted per gram of wet glandular tissue was calculated, and the result was expressed as μg of Alcian Blue/g of tissue.

#### Investigation of the role of sulfhydryl compounds (–SH groups) in gastroprotection

After fasting for 18 h, rats were distributed into six groups (n = 5–7/group). Three groups received 0.9% NaCl solution (control, 10 mL/kg, i.p.), and three groups received NEM (10 mg/kg, i.p.), which is a sulfhydryl compound blocker [17], to investigate the involvement of endogenous–SH groups in the gastroprotective effect of YMJ. After 30 min, the three groups that received 0.9% NaCl and the three groups that received NEM were pretreated as follows: one group received 0.9% NaCl solution by oral route, the second group was orally administered carbenoxolone (100 mg/kg), and the third group was orally administered 100% YMJ. One hour after pretreatment, gastric lesions were induced in all groups by oral administration of absolute ethanol (4 mL/kg). After 1 h, animals were euthanized, stomachs were removed and photographed, and the injuries were evaluated (Image J software, Bethesda, MD, USA). Results were expressed as the total area of ulcerative lesions (mm^2^).

#### Investigation of the role of prostaglandins in gastroprotection

After fasting for 18 h, rats were distributed into six groups (n = 4–8/group). Three groups received 0.9% NaCl solution (control, 10 mL/kg, i.p.), and three groups received indomethacin (30 mg/kg, i.p.), an NSAID that inhibits cyclooxygenase enzyme and thus, reduces the production of prostaglandins [17], to investigate the involvement of prostaglandins in the gastroprotective effect of YMJ. After 30 min, the three groups that received 0.9% NaCl and the three groups that received indomethacin were pretreated as follows: one group received 0.9% NaCl solution by oral route, the second group was orally administered misoprostol (50 μg/kg), and the third group was orally administered 100% YMJ (10 mL/kg). One hour after pretreatment, gastric lesions were induced in all groups by oral administration of absolute ethanol (4 mL/kg). Again, animals were euthanized 1 h after ethanol administration, stomachs were removed and photographed, and the injuries were evaluated by computerized planimetry using Image J software (Bethesda, MD, USA). Results were expressed as the total area of ulcerative lesions (mm^2^).

#### Determination of gastric acid secretion

This experiment was carried out according to the pylorus ligation method described by Shay et al. [18] with slight modifications. Animals were divided into four groups (n = 5–6/group): control, ranitidine, YMJ, and false-operated (received no treatment at all). After fasting for 18 h with free access to 5% glucose solution, animals were anesthetized by an i.p. injection of xylazine (6 mg/kg) and ketamine (60 mg/kg), then they were submitted to longitudinal incision for pylorus ligation. Immediately after ligation, animals were distributed into four groups (n = 5-6/group) as follows: control, rats received 0.9% NaCl (0.1 mL/100 g) by intraduodenal route; ranitidine, rats were administered ranitidine (60 mg/kg) by intraduodenal route; YMJ (100), rats were administered 100% YMJ (10 mL/kg) by intraduodenal route, and a false-operated group, which received no treatment at all. The abdominal wall was sutured, and 4 h later, animals were euthanized. Gastric secretions were collected and centrifuged at 176 × *g* for 30 min. Then, the gastric content (g), its pH, and the total acidity (mEquiv.[H^+^]/g/4 h) were determined.

### Evaluation of ulcer healing property of YMJ

#### Acetic acid-induced gastric ulcer

Chronic ulcer induction was carried out based on the method described by Takagi et al. [19] with some modifications. Animals were divided into three groups (n = 5–6/group), and the study lasted for 14 days. After fasting for 16 h, animals were anesthetized by an i.p. injection of xylazine (6 mg/kg) and ketamine (60 mg/kg) for surgical exposure of the stomach. Then, 0.05 mL of 30% acetic acid was injected into the subserosal layer of the external wall of the stomach. You can add the distribution here as follows: One day after the surgery, daily treatment began, and animals were distributed into three groups (n = 5-6/group) as follows: control, rats received 0.9% NaCl by oral route; ranitidine, rats were orally administered ranitidine (60 mg/kg); and YMJ (100), rats were orally administered 100% YMJ (10 mL/kg), once a day for 14 consecutive days. During the treatment, animals were observed for signs of toxicity, such as piloerection, diarrhea, changes in locomotor activity, or mortality. Moreover, body weight was recorded. On day 15, rats were euthanized, stomachs were removed and photographed, and the injuries were evaluated (Image J software, Bethesda, MD, USA). Results were expressed as the total area of ulcerative lesions (mm^2^).

In order to reduce the number of animals, our experimental procedure of gastric cicatrization using acetic acid followed the Russell-Burch Principles of reduction, substitution, and refinement in animal use (known as the 3R’s Principle). The experiments for this study and a related study were carried out jointly so that for the control and ranitidine groups, data from the same animals is used for both this study and a previously published study [9], allowing a reduction of 12 animals.

#### Histological analysis

Stomach tissue specimens with chronic ulcers (from acetic acid-induced rats) were sectioned and fixed in 10% buffered formalin. Then, tissue specimens were washed with water, immersed in 70% ethyl alcohol for 3–4 days, and embedded in paraffin. Finally, 5-μm thick paraffin sections were cut and stained with hematoxylin and eosin (H&E) and Periodic Acid–Schiff (PAS). Stained gastric tissue sections were examined under a light microscope and photographed using Leica camera, model EC3, attached to the microscope and connected to the Leica Application Suite software EZ (Leica, Wetzlar, Germany).

#### Immunohistochemical analysis

The immunohistochemical staining of Ki-67 and BrdU was carried out in 4-μm thick sections cut from paraffin blocks of samples containing ulcerated areas. Initially, samples were deparaffinized in xylene and hydrated [20].

The expression of Ki-67 and BrdU proteins was evaluated using the free-biotin method in conjugation with horseradish peroxidase (HRP). Antigen retrieval was performed using a pressure cooker for 2 min, after which the slides were cooled to room temperature, and endogenous peroxidase was blocked using bovine serum albumin for 1 h. After cooling, slides were incubated overnight with primary mouse monoclonal anti-Ki-67 antibody (Santa Cruz Biotechnology, code: sc-23900, dilution 1:200) and anti-BrdU antibody (Santa Cruz Biotechnology, IIB5, code: sc-32323, dilution 1:200). Visualization was carried out using HRP. Slides were washed, incubated with diaminobenzidine (DAB) chromogen solution (Dako Corporation, Carpiteria, CA USA), washed in water, counterstained with hematoxylin, dehydrated, and mounted. Cells positive for Ki-67 and BrdU were detected by the presence of a dark reddish-brown chromogen in the nucleus or nucleus/cytoplasm, respectively, of epithelial cells in the lesion area. Reactivity was scored using the following system: positive, mild (detected in 10–15% of the analyzed cells), moderate (in 25–50% of the analyzed cells), strong (in more than 50% of the analyzed cells); or negative (in less than 10% of the analyzed cells).

### Statistical analysis

Results were expressed as mean ± standard error of the mean (S.E.M). The differences between means were analyzed by analysis of variance (ANOVA) followed by Tukey’s test for one-way analysis. Statistical analysis was performed using GraphPad Prism 6.0^®^. The level of significance for rejection of the null hypothesis was set at 5% (p < 0.05).

## Results

### Phytochemical characterization of YMJ by HPLC

The manual extraction of juice from 5000 g of yellow mombin was able to yield approximately 400 mL of YMJ. A 2-mL aliquot was frozen, lyophilized, and the lyophile (0.48 g) was used for the phytochemical analysis. From the chromatographic analysis of YMJ, it was possible to identify and quantify two components by the similarity of their retention times and ultraviolet absorption spectrum (λ = 340 nm) to those of the analytical standards used: epicatechin at 20.2 min (7.1 ± 1.6 μg/mL), and quercetin at 14.5 min (17.3 ± 2.5 μg/mL).

### Gastroprotective activity of YMJ

#### Ethanol-induced gastric lesions

**Fig 1:**
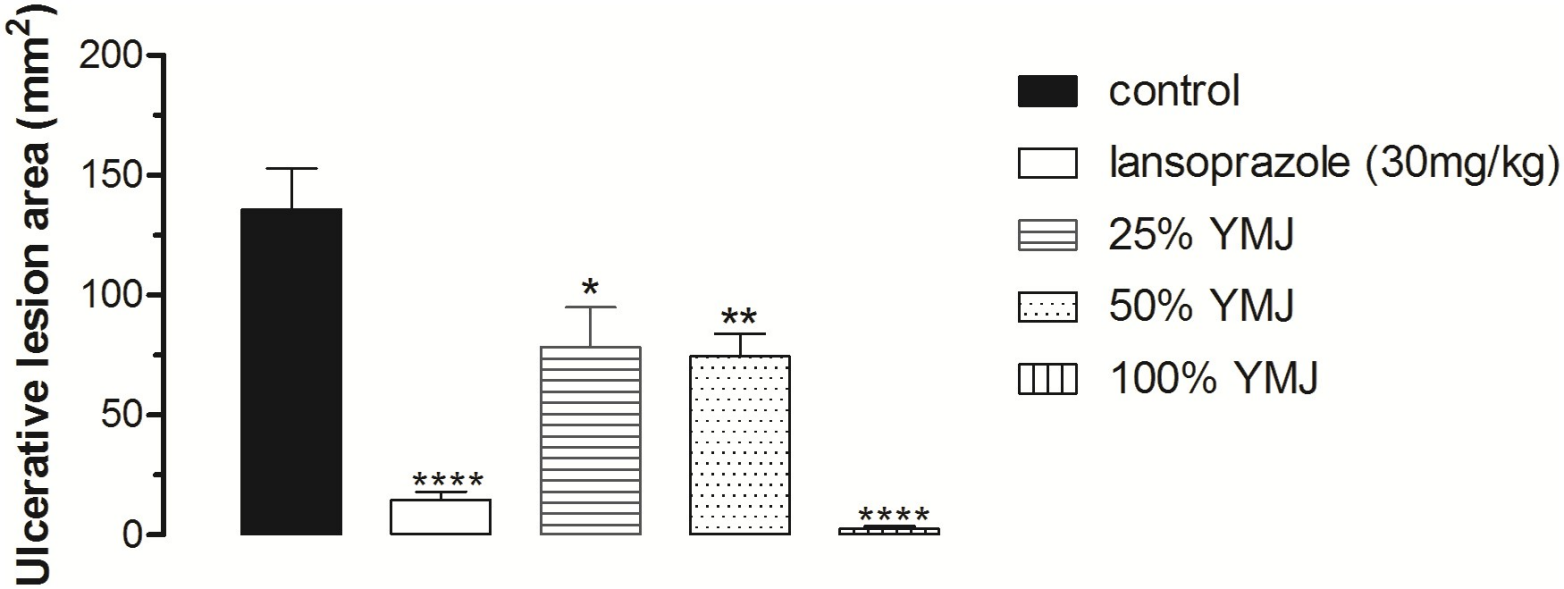
Effect of pretreatment with yellow mombin juice (YMJ) on ethanol-induced gastric lesions in rats. Results are expressed as mean ± S.E.M. (n = 6/group). Results were analyzed by one-way ANOVA followed by Tukey’s test, *p < 0.05, **p < 0.01, ****p < 0.0001.

**Fig 2:**
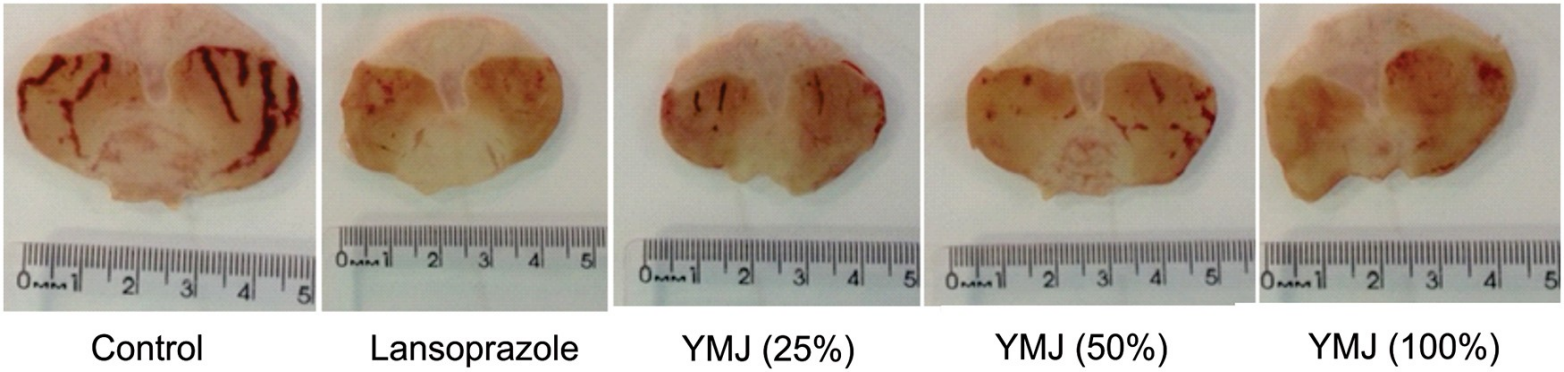
Photomicrographs of stomach sections from rats with ethanol-induced gastric lesions.

Ethanol administration resulted in extensive damage to the gastric mucosa with hemorrhagic erosions in the control group (pretreated with 0.9% NaCl). However, pretreatment with YMJ (25, 50, and 100%) and lansoprazole significantly inhibited the gastric lesions by 42.42, 45.09, 98.21, and 89.22%, respectively, compared to the control group (Figs 1 and 2).

#### Indomethacin-induced gastric lesions

**Fig 3:**
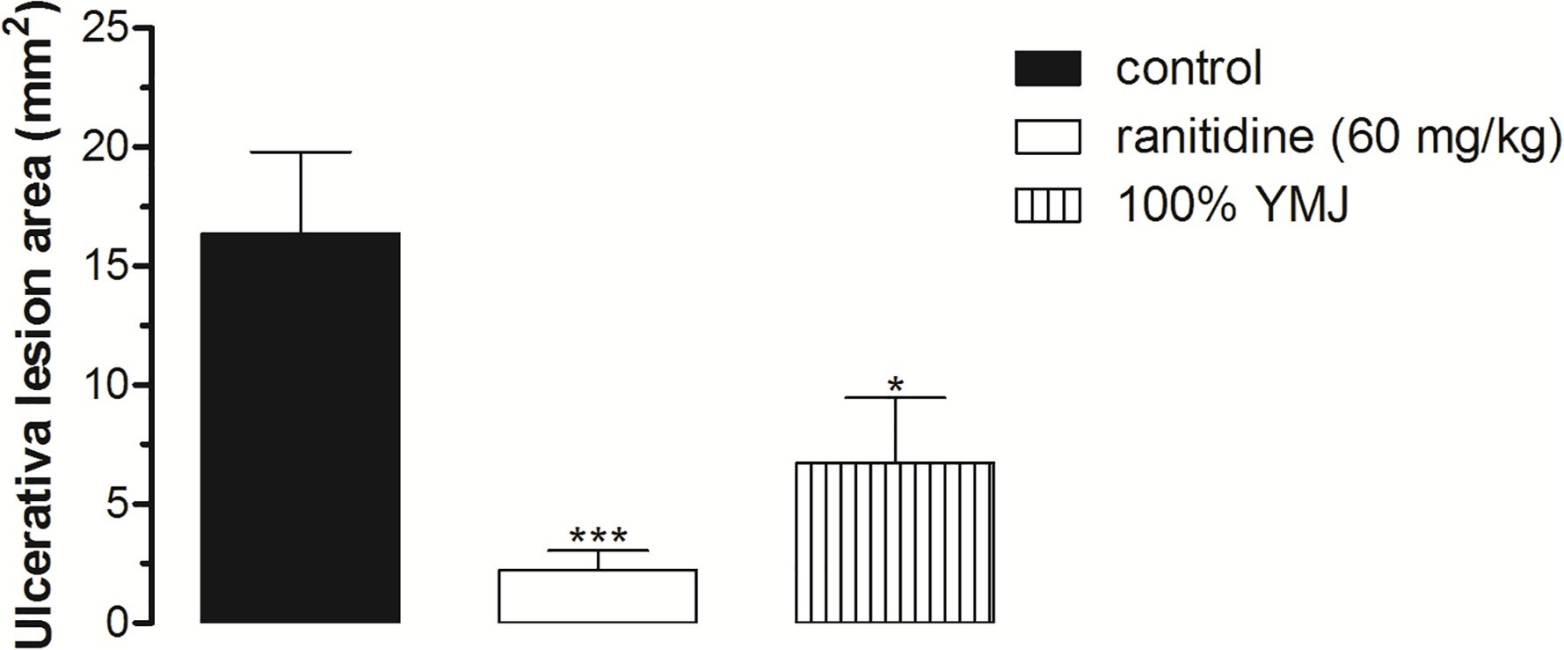
Effect of pretreatment with yellow mombin juice (YMJ) on indomethacin-induced gastric lesions in rats. Results are expressed as mean ± S.E.M. (n = 6–8/group). Results were analyzed by one-way ANOVA followed by Tukey’s test, *p < 0.05, ***p < 0.001.

**Fig 4:**
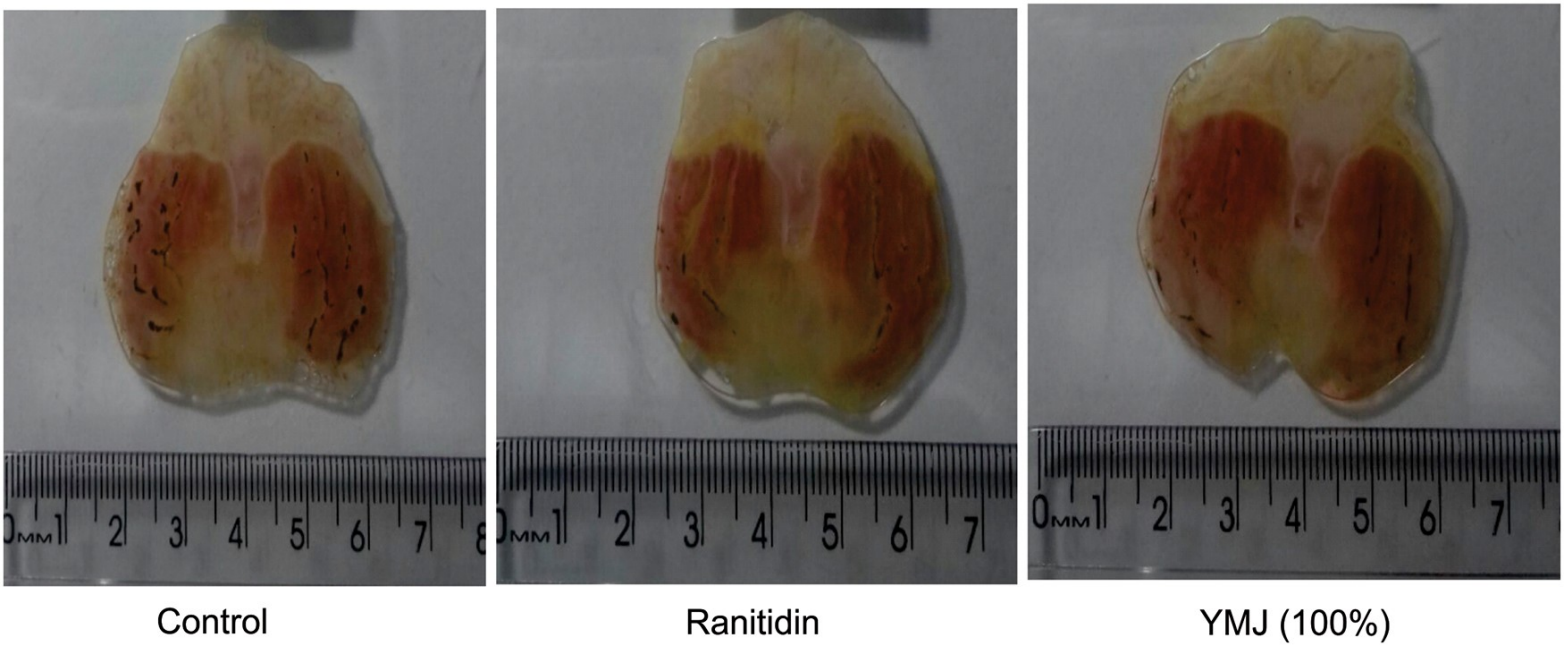
Photomicrographs of stomach sections from rats with indomethacin-induced gastric lesions.

The subcutaneous administration of indomethacin (30 mg/kg) resulted in injury to the gastric mucosa with an area of 16.36 ± 3.44 mm^2^ in the control group. Pretreatment with pure YMJ (100%) and ranitidine (60 mg/kg) significantly inhibited the gastric lesions by 58.96% and 86.43%, respectively, compared to the control group (Figs 3 and 4).

### Evaluation of mucosal protective factors

#### Effect of yellow mombin juice (YMJ) on the production of gastric mucus

**Fig 5:**
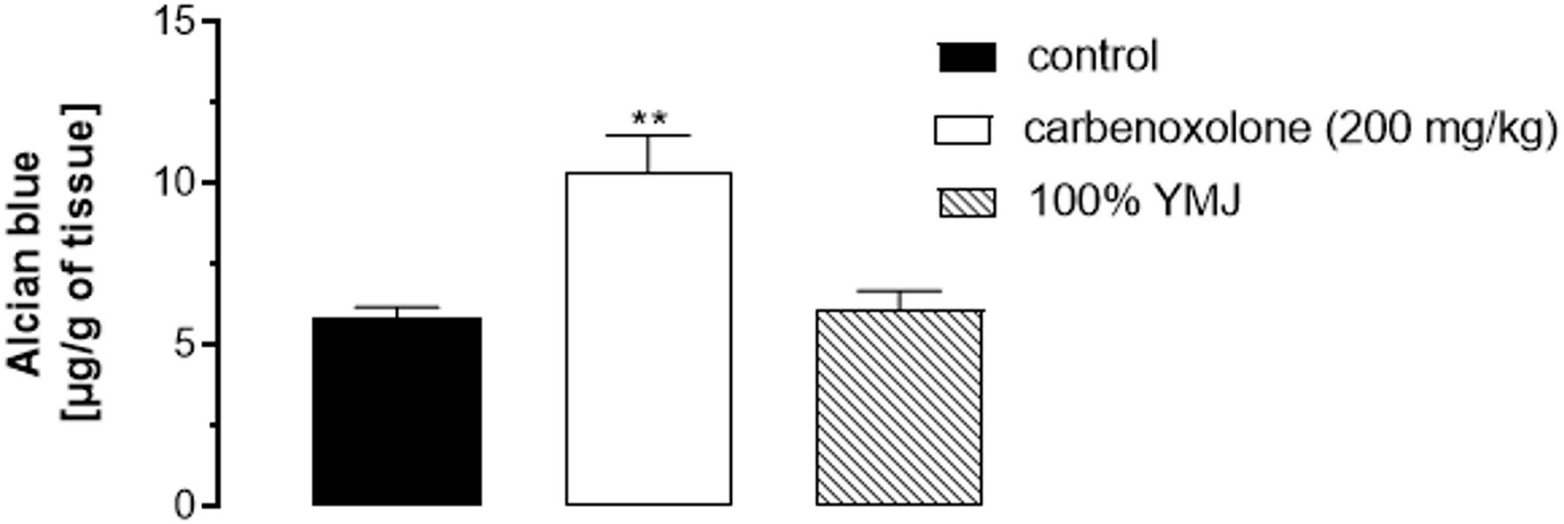
Effect of pretreatment with yellow mombin juice (YMJ) on gastric mucus production. Results are expressed as mean ± S.E.M. (n = 6/group). Results were analyzed by one-way ANOVA followed by Tukey’s test, **p < 0.01.

The gastric mucus level in control group animals submitted to pylorus ligation was found to be 8.79 ± 0.67 μg of Alcian Blue/g of tissue. Pretreatment with carbenoxolone (200 mg/kg) significantly increased the production of mucus by 76.8%, while pretreatment with YMJ did not significantly alter the mucus production, compared to the injured control group (Fig 5).

#### The role of sulfhydryl compounds (–SH groups) in gastroprotection

**Fig 6:**
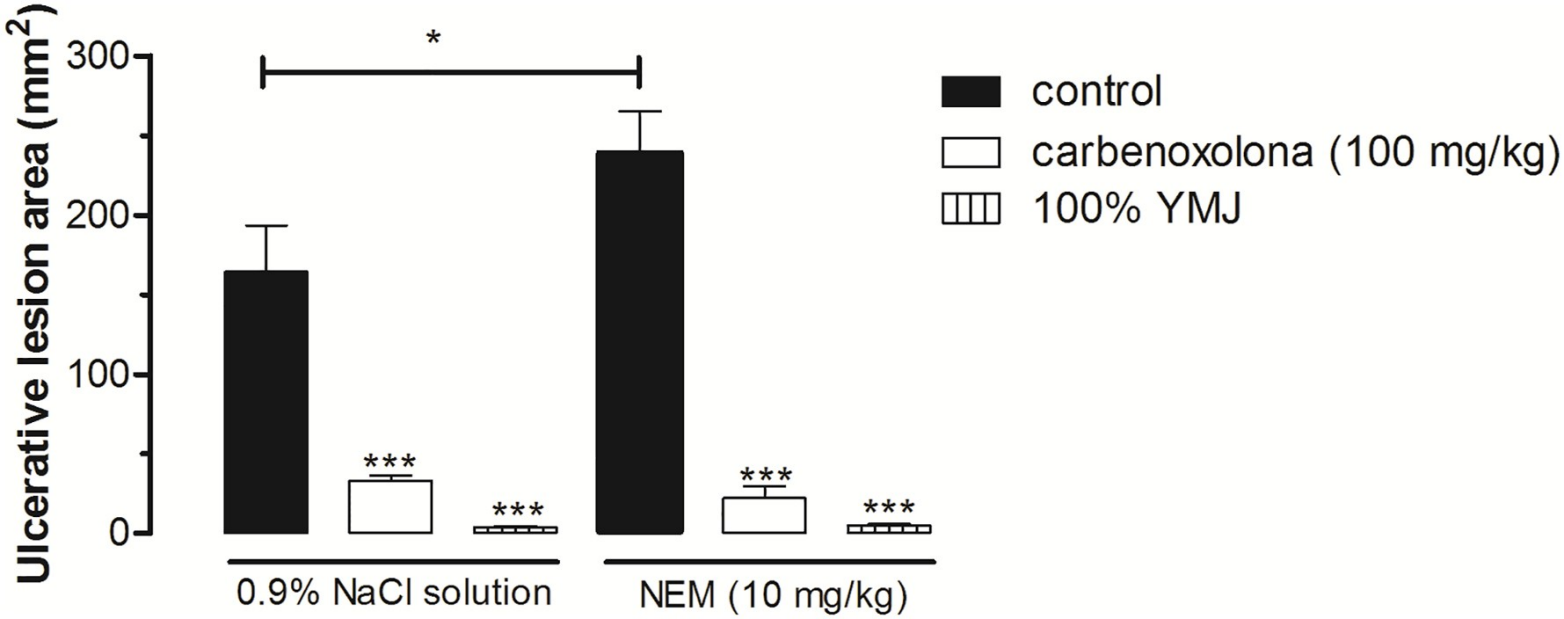
Effect of yellow mombin juice (YMJ) on gastric lesions induced by ethanol in N-ethylmaleimide (NEM)-administered rats. Results are expressed as mean ± S.E.M. (n = 5–7/group). Results were analyzed by one-way ANOVA followed by Tukey’s test, *p < 0.05, ***p < 0.001.

Administration of NEM exacerbated ethanol-induced gastric lesions by 45.38% compared to the groups administered NaCl solution. However, pretreatment with YMJ (100%) and carbenoxolone (100 mg/kg) significantly inhibited the ulcerative lesions induced by absolute ethanol in both NaCl and NEM-administered groups, i.e., YMJ continued to exert its gastroprotective effect even after NEM administration (Fig 6).

#### The role of prostaglandins in gastroprotection

**Fig 7:**
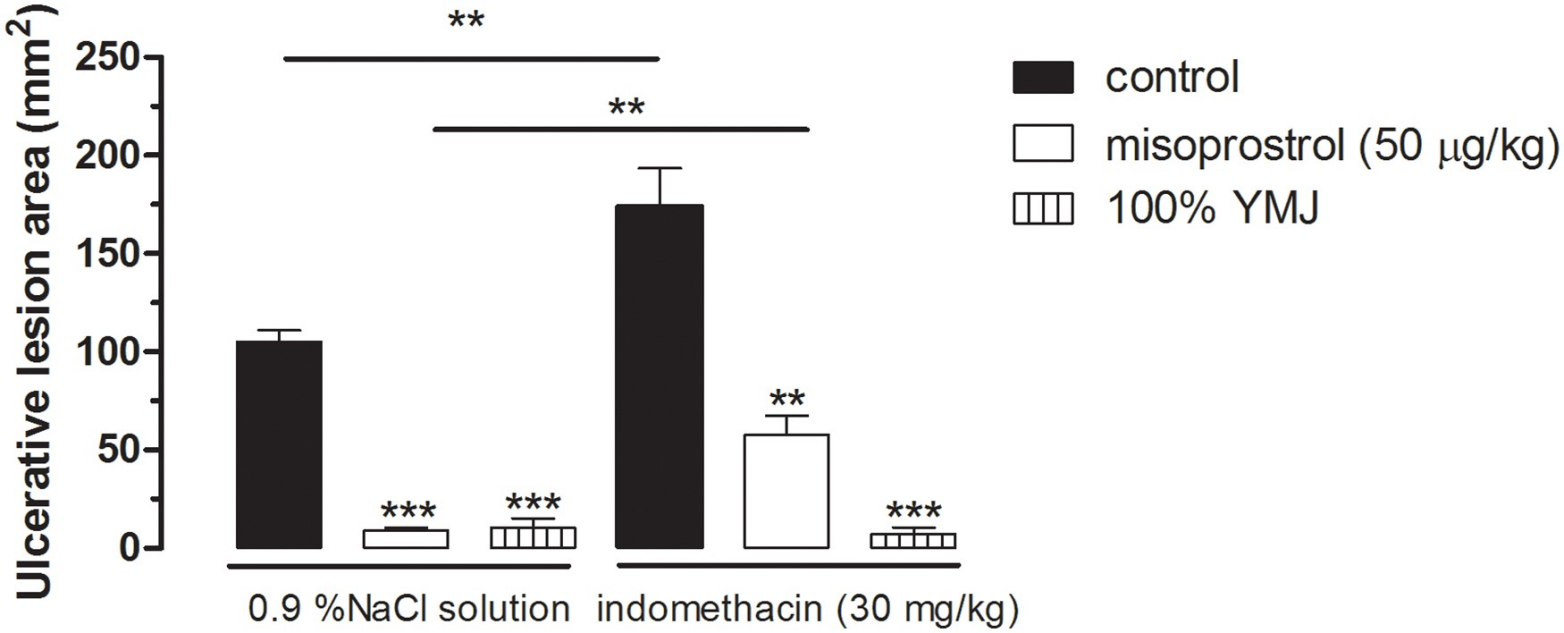
Effect of yellow mombin juice (YMJ) on gastric lesions induced by ethanol in indomethacin-administered rats. Results are expressed as mean ± S.E.M. (n = 4–8/group). Results were analyzed by one-way ANOVA followed by Tukey’s test, **p < 0.01, ***p < 0.001.

Administration of indomethacin exacerbated ethanol-induced gastric lesions in groups pretreated with NaCl and misoprostol by 65.54% and 553.08%, respectively, compared to the NaCl-administered groups pretreated with NaCl and misoprostol, respectively. However, pretreatment with YMJ (100%) significantly inhibited the ulcerative lesions induced by absolute ethanol in both NaCl and indomethacin-administered groups, with no significant difference between NaCl-administered/YMJ-pretreated and indomethacin-administered/YMJ-pretreated groups (Fig 7).

#### Effect of YMJ on gastric acid secretion

Four hours after pylorus ligation and intraduodenal administration of NaCl, ranitidine, and YMJ, animals were euthanized and gastric secretions were collected, It was found that the intraduodenal administration of YMJ (100%) and ranitidine (60 mg/kg) significantly reduced the total acidity by 71.97 and 51.50%, respectively, and the content of gastric secretions by 57.35 and 66.17%, respectively (Table 1).

**Table 1:** Effect of intraduodenal administration of yellow mombin juice (YMJ) on gastric secretion in Wistar rats subjected to pylorus ligation.

Treatment	pH	Total acidy (mEquiv.[H^+^]/g/4 h)	Gastric content (g)
False-operated	5.33 ± 0.31**	10.78 ± 0.85**	0.16 ± 0.01***
Control	3.06 ± 0.08	17.30 ± 1.42	0.68 ± 0.01
100% YMJ (10 mL/kg)	3.45 ± 0.31	4.85 ± 1.04***	0.29 ± 0.11**
Ranitidine (60 mg/kg)	3.95 ± 0.11*	8.39 ± 0.89***	0.23 ± 0.03***

Results are expressed as mean ± S.E.M. (n = 5–6/group). Results were analyzed by one-way ANOVA followed by Tukey’s test,

*p < 0.05,

**p < 0.01,

***p < 0.001.

### Ulcer healing properties yellow mombin juice (YMJ)

#### Acetic acid-induced gastric ulcer

**Fig 8:**
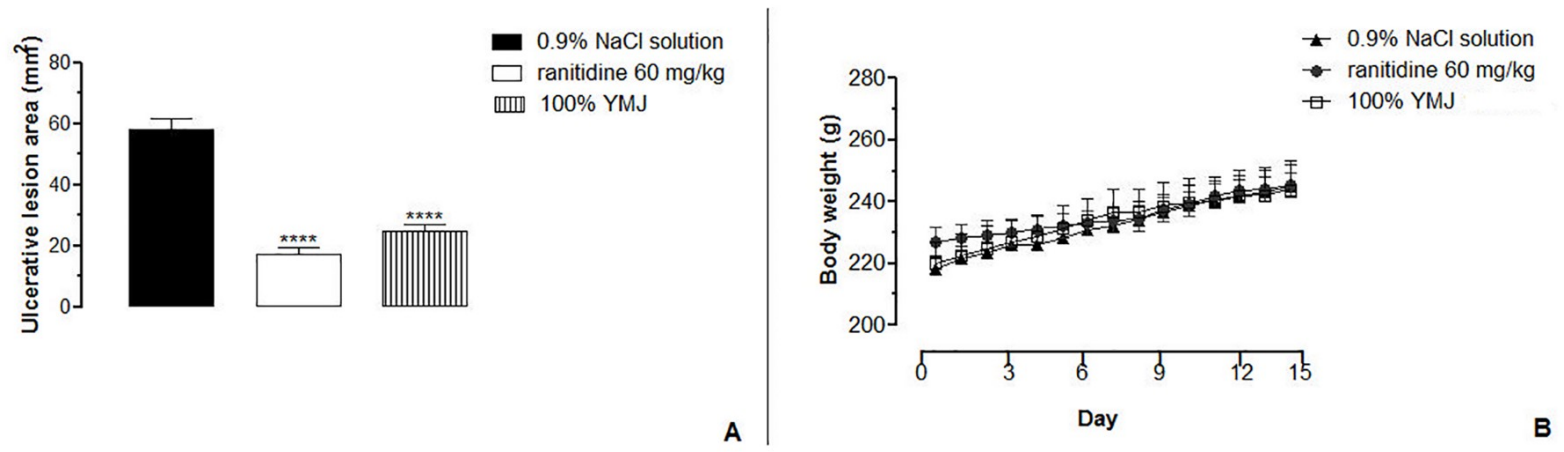
Effect of yellow mombin juice (YMJ) on healing of the gastric mucosa in rats with chronic acetic acid-induced gastric ulcer. Results are expressed as mean ± S.E.M. (n = 5–6/group). Results were analyzed by one-way ANOVA followed by Tukey’s test, ****p < 0.0001.

Results of this experiment showed that treatment with YMJ (100%) and ranitidine (60 mg/kg) for 14 consecutive days decreased the area of the chronic ulcer by 57.34 and 70.65%, respectively, compared to the control group, which demonstrated an ulcer area of 58.19 ± 3.29 mm^2^ (Fig 8). These results demonstrate that YMJ has a healing effect on chronic acetic acid-induced ulcers (Fig 8A). Furthermore, throughout the study period, daily administration of YMJ caused no sign of visible toxicity, death, or alteration in body mass (Fig 8B).

**Fig 9:**
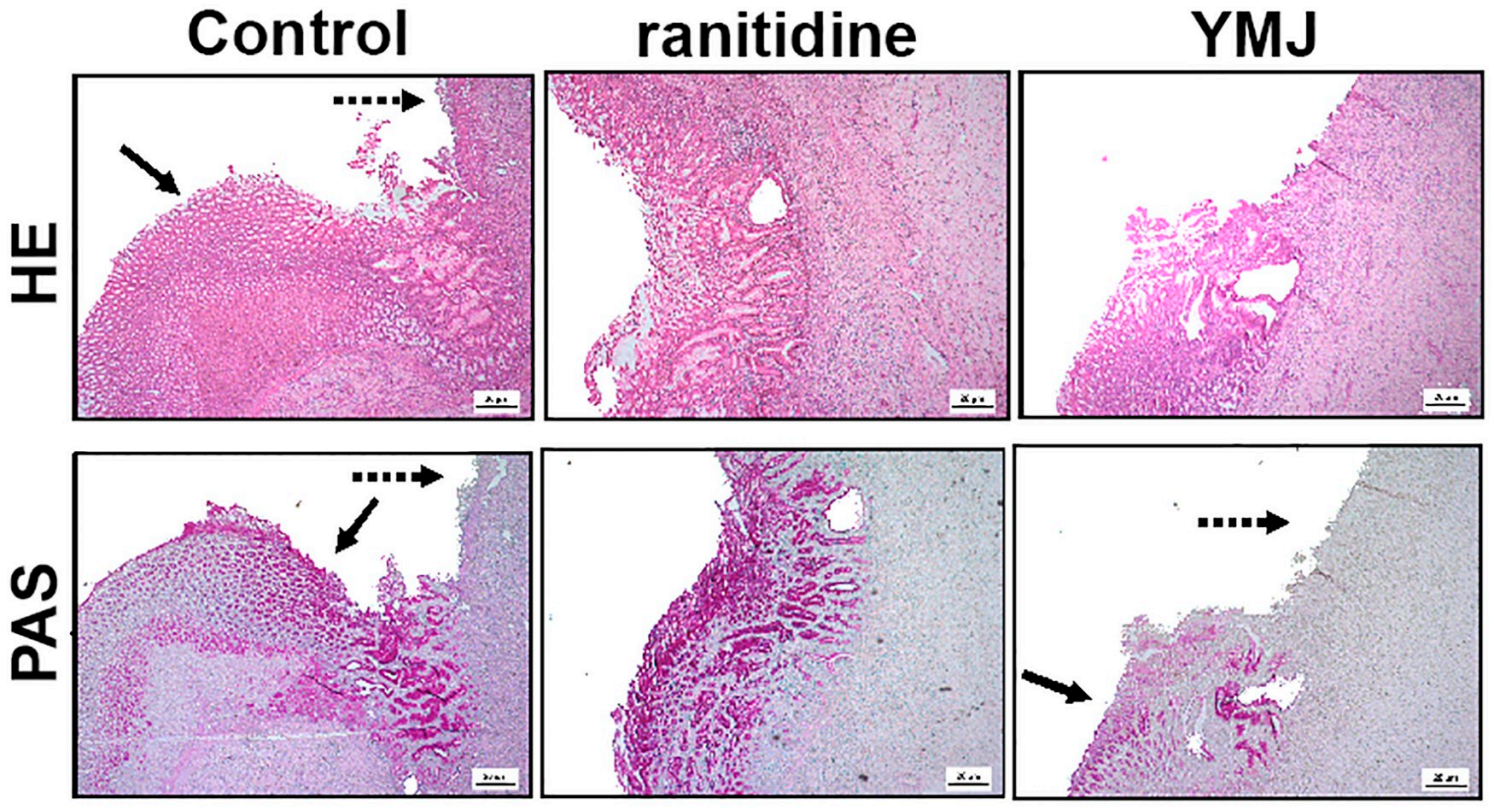
Photomicrographs of gastric mucosa sections stained with H&E and PAS from rats with chronic acetic acid-induced gastric ulcer. Animals were orally treated with 0.9% NaCl solution (control group), ranitidine (60 mg/kg), or YMJ (100%) for 14 days. The filled arrow indicates the absence of the epithelial layer (internal ulcer area), and the dashed arrow indicates the remaining epithelial layer (ulcer edge). H&E, Hematoxylin and eosin; PAS, Periodic Acid–Schiff; magnification, 40x.

Histological analysis of H&E-stained stomach specimens revealed well-defined ulcers with complete destruction of the mucosal and submucosal layers caused by acetic acid administration in control group rats. However, stomach specimens from rats orally treated with YMJ (10 mL/kg) and ranitidine (60 mg/kg) demonstrated regeneration of the gastric mucosa, as revealed by the reappearance of the epithelial and stromal layers, compared to the control group. Moreover, PAS staining showed increased mucus production, as demonstrated by the areas intensely stained by magenta color in the mucosal epithelial layer (Fig 9).

**Fig 10:**
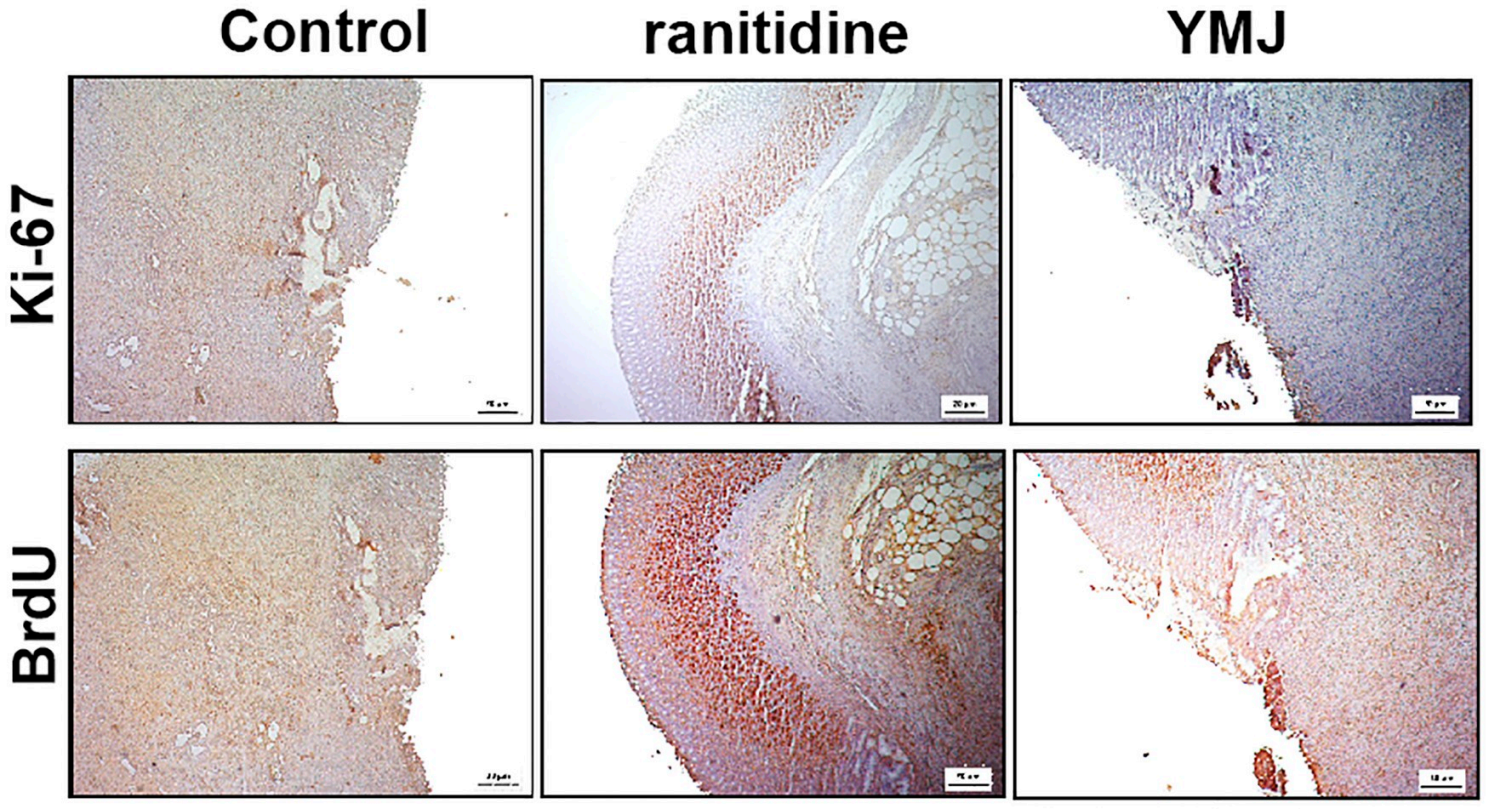
Immunohistochemical staining of Ki-67 and BrdU in the gastric mucosa of rats with chronic acetic acid-induced gastric ulcer. Animals were orally treated with 0.9% NaCl solution (control group), ranitidine (60 mg/kg), or YMJ (100%) for 14 days. The filled arrow indicates the absence of the epithelial layer (internal ulcer area), and the dashed arrow indicates the remaining epithelial layer (ulcer edge). Photomicrographs demonstrate the immunoreactivity to Ki-67 and BrdU, magnification, 200x.

Immunohistochemical investigation using monoclonal antibodies against Ki-67 and BrdU showed strong reactivity to BrdU, marked by the great quantity of BrdU-positive nuclei, and moderate reactivity to Ki-67, as demonstrated by the dark reddish-brown color in the gastric mucosa of animals treated with YMJ and ranitidine for 14 days. On the other hand, tissues from the control group demonstrated no reactivity for any of the two markers due to the destruction of the epithelial layer, as shown in Fig 10.

## Discussion

This study was carried out to investigate the gastroprotective activity of YMJ, the juice of *S*. *mombin* fruit, in acute gastric lesions induced by different necrotizing agents, and its healing effect in chronic gastric lesions induced by acetic acid, as well as the mechanisms involved in its action.

It was found that induction of gastric lesions by ethanol results in depletion of gastric defensive mechanisms, starting from the formation of reactive oxygen species (ROS) such as superoxide anions, hydroxyl radicals, and lipid peroxides [21] that result in glutathione depletion and thus, intracellular oxidative stress, changes in membrane permeability, and mitochondrial membrane depolarization that ultimately lead to cell death [22]. Our results showed that administration of pure (100%) and diluted (50 and 25%) YMJ to ethanol-induced rats significantly inhibited ethanol-induced gastric injury.

Administration of indomethacin was found to suppress prostaglandin synthesis and thus, increase susceptibility to gastric mucosal lesions, as shown in the control group. However, pretreatment with YMJ significantly reduced mucosal damage compared to the respective control group pretreated with NaCl.

Quercetin, a compound found in YMJ, was previously reported to inhibit the production of ROS and act as an anti-apoptotic during gastric injury [23]. Moreover, quercetin, in combination with pantoprazole (a proton pump inhibitor), was reported to prevent diclofenac sodium-induced gastroenteropathy in rats [24]. Epicatechin, another compound found in YMJ, is a polyphenolic compound that was reported by Rozza et al. [25] to have gastroprotective properties and to strengthen the mucus barrier, neutralize the gastric juice, and act as an antioxidant. HPLC results identified the presence of quercetin and epicatechin as major compounds in YMJ, which suggests that, at least in part, the gastroprotective effects of YMJ are mediated by these compounds.

From our results, YMJ was found to possess gastroprotective properties in ethanol and indomethacin models of acute gastric injury, which suggests the potential involvement of prostaglandins, mucus production, and/or antioxidant capacity in the antiulcer activity of YMJ [26].

As far as we know, this is the first study to investigate the gastroprotective effect of the juice from the fruit pulp of *S*. *mombin*. Tiburski et al. [27] reported the nutritional composition and *in vitro* antioxidant capacity of the frozen pulp of yellow mombin. The antioxidant activity was determined by the Trolox equivalent antioxidant capacity assay using the radical cation ABTS^+^ (2,2-azobis-(3-wtilbenzotiazolina-6-sulfonato)., and was found to be 17.47 ± 3.27 mmol Trolox equivalent/g. Data from the literature suggest that agents that increase the antioxidant defenses of the gastric mucosa are considered to be beneficial in the treatment of gastric lesions [20, 28].

After confirming the gastroprotective action of the juice, we aimed to elucidate the possible mechanisms of this action through a number of experiments. First, the effect of YMJ on mucus production was evaluated. As expected, carbenoxolone increased mucus production; however, YMJ did not affect it. This result negates the enhancing effect of YMJ on mucus production. Increased mucus secretion is among the important mechanisms of gastric mucosal defense against necrotizing agents [29, 30]. In addition, when associated with bicarbonate production, it may play a significant role in the prevention of ulcer formation and the protection of newly formed cells from acid and peptic injury [17].

Then, the roles of–SH groups and prostaglandins in the mechanism of YMJ action were investigated. Our results showed that there was no significant difference between the gastroprotective effect of YMJ in the absence and presence of NEM, as well as in the absence and presence of indomethacin. These data indicate that the gastroprotective effect of YMJ is not associated with the production and/or presence of sulfhydryl compounds and does not depend on the production of prostaglandins. The–SH groups are responsible for increasing the production and maintaining the stability of gastric mucus through the formation of disulfide bridges, and are involved in maintaining gastric integrity by limiting the production of free radicals involved in tissue damage [31]. The relatively high concentration of–SH groups in the gastric mucosa indicates their possible role in gastroprotection [3, 20].

Prostaglandins may be involved in the gastroprotection process by inhibiting gastric acid secretion or enhancing gastric mucosal protective factors such as stimulation of mucus secretion, bicarbonate, and increased membrane phospholipids [32].

After pylorus ligation, the intraduodenal administration of YMJ was effective at reducing the concentration of H^+^ ions, thereby decreasing the gastric acidity, and also decreasing the content of gastric secretions. These results indicate that the antisecretory activity of YMJ could contribute to its gastroprotective activity. In addition, data from the literature [33, 34] show that catechin and epicatechin are potent non-competitive inhibitors of H^+^/K^+^-ATPase, which supports the antisecretory activity of YMJ demonstrated in this study.

Moreover, YMJ assisted the healing of acetic acid-induced gastric injury in rats. The acetic acid model highly resembles human peptic ulcer regarding pathological features, the healing process, and the ulcer recurrence cycle [35]. Thus, it is largely applied to evaluate the gastric healing capacity of synthetic or phytochemical substances [20, 36, 37].

Administration of YMJ once daily for 14 days after the induction of gastric injury with acetic acid accelerated the healing process through mucosal and submucosal re-epithelialization, as demonstrated by H&E staining. Moreover, the YMJ-treated group was found to restore mucus production in glandular mucus producing cells, as demonstrated by PAS staining. The mucus layer acts as the first line of defense against luminal pepsin by forming a pre-epithelial mucus-buffer barrier along with phospholipids [38].

Immunohistochemical analysis was used to verify the regenerative capacity of YMJ on the gastric mucosa through the detection of proliferating cells at the site of the chronic ulcer. Ki-67 is a nuclear matrix protein expressed by proliferating cells during the G1, S, G2, and M phases of the cell cycle, but not in quiescent cells [39], while BrdU is a synthetic nucleoside of thymidine that is incorporated into DNA during the S phase of the cell cycle and is expressed by proliferating cells [40, 41]. Cell proliferation plays an important role in wound healing. Our results suggest that the antiulcerogenic capacity of YMJ could be mediated by enhancing cell proliferation and thus, increasing the expression of Ki-67 and BrdU in the process of gastric healing. In fact, the effectiveness of Ki-67, BrdU, and proliferating cell nuclear antigen as markers of cellular proliferation was recently reported by Caldas et al. [20] as part of the strategy to evaluate the gastric healing capacity of 1,8-cineole in Wistar rats.

## Conclusion

Yellow mombin juice (YMJ) was found to exhibit a significant gastroprotective activity, the mechanism of which may be associated with its antisecretory action. The juice was found to be effective in accelerating the process of re-epithelialization in rats. Findings of this study open the perspective for the exploitation of YMJ as a functional food.
